# Supplementary material for: Optimal Body Mass Index Cut-off Point for Predicting Colorectal Cancer Survival in an Asian Population: A National Health Information Database Analysis
Source: Cancers (Basel). 2020 Mar 30;12(4):830. doi: 10.3390/cancers12040830 (PMC7226173; doi:10.3390/cancers12040830)
Supplement: Supplementary file 1 [file cancers-12-00830-s001.pdf]

Supplementary Materials: Optimal Body Mass Index Cut-off Point for Predicting Colorectal Cancer Survival in an Asian Population: A National Health Information Database Analysis

Nan Song, Dan Huang, Doeun Jang, Minjung Kim, Seung-Yong Jeong, Aesun Shin and Ji Won Park

Table S1. Estimation of potential BMI cut-off point for overall survival by Contal and O'Quigley's method.

No.	BMI	L_k	$\lvert L_k \rvert$	Q-statistic	*p*
1	13.00	0.00	0.00	0.00	0.3000
2	13.10	−1.00	1.00	0.03	0.3000
3	13.20	−1.00	1.00	0.03	0.3000
4	13.30	−1.00	1.00	0.03	0.3000
5	13.40	−1.00	1.00	0.03	0.3000
6	13.50	−1.00	1.00	0.03	0.3000
7	13.60	−1.00	1.00	0.03	0.3000
8	13.70	−1.96	1.96	0.06	0.3000
9	13.80	−1.96	1.96	0.06	0.3000
10	13.90	−3.91	3.91	0.12	0.3000
11	14.00	−5.75	5.75	0.18	0.3000
12	14.10	−5.75	5.75	0.18	0.3000
13	14.20	−5.75	5.75	0.18	0.3000
14	14.30	−6.62	6.62	0.21	0.3000
15	14.40	−6.62	6.62	0.21	0.3000
16	14.50	−6.62	6.62	0.21	0.3000
17	14.60	−8.16	8.16	0.26	0.3000
18	14.70	−8.16	8.16	0.26	0.3000
19	14.80	−8.16	8.16	0.26	0.3000
20	14.90	−8.16	8.16	0.26	0.3000
21	15.00	−7.92	7.92	0.25	0.3000
22	15.10	−8.62	8.62	0.27	0.3000
23	15.20	−9.27	9.27	0.29	0.3000
24	15.30	−10.17	10.17	0.32	0.3000
25	15.40	−9.87	9.87	0.31	0.3000
26	15.50	−10.75	10.75	0.34	0.3000
27	15.60	−12.46	12.46	0.40	0.3000
28	15.70	−12.46	12.46	0.40	0.3000
29	15.80	−12.29	12.29	0.39	0.3000
30	15.90	−13.04	13.04	0.41	0.3000
31	16.00	−14.03	14.03	0.45	0.3000
32	16.10	−13.87	13.87	0.44	0.3000
33	16.20	−17.91	17.91	0.57	0.3000
34	16.30	−20.22	20.22	0.64	0.3000
35	16.40	−21.73	21.73	0.69	0.3000
36	16.50	−22.08	22.08	0.70	0.3000
37	16.60	−24.82	24.82	0.79	0.3000
38	16.70	−26.56	26.56	0.84	0.3000
39	16.80	−27.58	27.58	0.88	0.3000
40	16.90	−27.90	27.90	0.89	0.3000
41	17.00	−30.11	30.11	0.96	0.3000
42	17.10	−34.84	34.84	1.11	0.1738

43	17.20	−36.14	36.14	1.15	0.1443
44	17.30	−37.27	37.27	1.18	0.1221
45	17.40	−38.32	38.32	1.22	0.1041
46	17.50	−39.58	39.58	1.26	0.0853
47	17.60	−44.31	44.31	1.41	0.0384
48	17.70	−44.45	44.45	1.41	0.0375
49	17.80	−47.16	47.16	1.50	0.0227
50	17.90	−50.57	50.57	1.60	0.0116
51	18.00	−48.29	48.29	1.53	0.0183
52	18.10	−50.62	50.62	1.61	0.0115
53	18.20	−53.47	53.47	1.70	0.0063
54	18.30	−50.93	50.93	1.62	0.0108
55	18.40	−49.83	49.83	1.58	0.0135
56	18.50	−51.52	51.52	1.63	0.0096
57	18.60	−55.95	55.95	1.78	0.0037
58	18.70	−61.52	61.52	1.95	0.0010
59	18.80	−65.33	65.33	2.07	0.0004
60	18.90	−66.36	66.36	2.11	0.0003
61	19.00	−70.27	70.27	2.23	<0.0001
62	19.10	−75.44	75.44	2.39	<0.0001
63	19.20	−77.83	77.83	2.47	<0.0001
64	19.30	−76.68	76.68	2.43	<0.0001
65	19.40	−75.42	75.42	2.39	<0.0001
66	19.50	−82.48	82.48	2.62	<0.0001
67	19.60	−82.01	82.01	2.60	<0.0001
68	19.70	−86.74	86.74	2.75	<0.0001
69	19.80	−84.37	84.37	2.68	<0.0001
70	19.90	−86.30	86.30	2.74	<0.0001
71	20.00	−86.27	86.27	2.74	<0.0001
72	20.10	−92.38	92.38	2.93	<0.0001
73	20.20	−96.69	96.69	3.07	<0.0001
74	20.30	−96.56	96.56	3.06	<0.0001
75	20.40	−93.93	93.93	2.98	<0.0001
76	20.50	−92.67	92.67	2.94	<0.0001
77	20.60	−94.91	94.91	3.01	<0.0001
78	20.70	−93.93	93.93	2.98	<0.0001
79	20.80	−91.27	91.27	2.90	<0.0001
80	20.90	−82.77	82.77	2.63	<0.0001
81	21.00	−78.75	78.75	2.50	<0.0001
82	21.10	−77.48	77.48	2.46	<0.0001
83	21.20	−72.07	72.07	2.29	<0.0001
84	21.30	−77.23	77.23	2.45	<0.0001
85	21.40	−76.13	76.13	2.42	<0.0001
86	21.50	−74.13	74.13	2.35	<0.0001
87	21.60	−77.26	77.26	2.45	<0.0001
88	21.70	−76.38	76.38	2.42	<0.0001
89	21.80	−83.78	83.78	2.66	<0.0001
90	21.90	−84.15	84.15	2.67	<0.0001
91	22.00	−79.06	79.06	2.51	<0.0001
92	22.10	−75.08	75.08	2.38	<0.0001
93	22.20	−73.09	73.09	2.32	<0.0001
94	22.30	−78.70	78.70	2.50	<0.0001
95	22.40	−84.62	84.62	2.68	<0.0001
96	22.50	−80.79	80.79	2.56	<0.0001
97	22.60	−78.24	78.24	2.48	<0.0001
98	22.70	−77.01	77.01	2.44	<0.0001
99	22.80	−71.79	71.79	2.28	<0.0001
100	22.90	−72.87	72.87	2.31	<0.0001

101	23.00	−74.17	74.17	2.35	<0.0001
102	23.10	−75.18	75.18	2.39	<0.0001
103	23.20	−74.12	74.12	2.35	<0.0001
104	23.30	−70.61	70.61	2.24	<0.0001
105	23.40	−70.75	70.75	2.24	<0.0001
106	23.50	−66.19	66.19	2.10	0.0003
107	23.60	−60.38	60.38	1.92	0.0013
108	23.70	−54.78	54.78	1.74	0.0048
109	23.80	−57.66	57.66	1.83	0.0025
110	23.90	−56.40	56.40	1.79	0.0033
111	24.00	−51.01	51.01	1.62	0.0106
112	24.10	−54.82	54.82	1.74	0.0047
113	24.20	−54.01	54.01	1.71	0.0056
114	24.30	−48.87	48.87	1.55	0.0163
115	24.40	−47.38	47.38	1.50	0.0218
116	24.50	−41.38	41.38	1.31	0.0637
117	24.60	−35.83	35.83	1.14	0.1508
118	24.70	−31.64	31.64	1.00	0.2665
119	24.80	−31.15	31.15	0.99	0.3000
120	24.90	−29.90	29.90	0.95	0.3000
121	25.00	−29.16	29.16	0.93	0.3000
122	25.10	−31.36	31.36	0.99	0.3000
123	25.20	−35.71	35.71	1.13	0.1535
124	25.30	−33.09	33.09	1.05	0.2207
125	25.40	−30.49	30.49	0.97	0.3000
126	25.50	−27.11	27.11	0.86	0.3000
127	25.60	−24.16	24.16	0.77	0.3000
128	25.70	−24.11	24.11	0.76	0.3000
129	25.80	−22.54	22.54	0.72	0.3000
130	25.90	−22.68	22.68	0.72	0.3000
131	26.00	−20.39	20.39	0.65	0.3000
132	26.10	−17.05	17.05	0.54	0.3000
133	26.20	−17.93	17.93	0.57	0.3000
134	26.30	−16.45	16.45	0.52	0.3000
135	26.40	−15.45	15.45	0.49	0.3000
136	26.50	−12.14	12.14	0.39	0.3000
137	26.60	−10.86	10.86	0.34	0.3000
138	26.70	−11.26	11.26	0.36	0.3000
139	26.80	−8.45	8.45	0.27	0.3000
140	26.90	−5.66	5.66	0.18	0.3000
141	27.00	−4.06	4.06	0.13	0.3000
142	27.10	−1.89	1.89	0.06	0.3000
143	27.20	−0.20	0.20	0.01	0.3000
144	27.30	1.26	1.26	0.04	0.3000
145	27.40	2.48	2.48	0.08	0.3000
146	27.50	7.01	7.01	0.22	0.3000
147	27.60	7.26	7.26	0.23	0.3000
148	27.70	6.73	6.73	0.21	0.3000
149	27.80	7.05	7.05	0.22	0.3000
150	27.90	7.33	7.33	0.23	0.3000
151	28.00	5.69	5.69	0.18	0.3000
152	28.10	5.23	5.23	0.17	0.3000
153	28.20	6.17	6.17	0.20	0.3000
154	28.30	9.50	9.50	0.30	0.3000
155	28.40	6.20	6.20	0.20	0.3000
156	28.50	7.84	7.84	0.25	0.3000
157	28.60	8.71	8.71	0.28	0.3000
158	28.70	9.26	9.26	0.29	0.3000

159	28.80	10.13	10.13	0.32	0.3000
160	28.90	10.25	10.25	0.33	0.3000
161	29.00	6.62	6.62	0.21	0.3000
162	29.10	4.83	4.83	0.15	0.3000
163	29.20	5.98	5.98	0.19	0.3000
164	29.30	4.15	4.15	0.13	0.3000
165	29.40	4.54	4.54	0.14	0.3000
166	29.50	3.12	3.12	0.10	0.3000
167	29.60	3.48	3.48	0.11	0.3000
168	29.70	4.42	4.42	0.14	0.3000
169	29.80	4.23	4.23	0.13	0.3000
170	29.90	5.84	5.84	0.19	0.3000
171	30.00	5.25	5.25	0.17	0.3000
172	30.10	6.44	6.44	0.20	0.3000
173	30.20	7.65	7.65	0.24	0.3000
174	30.30	6.49	6.49	0.21	0.3000
175	30.40	7.48	7.48	0.24	0.3000
176	30.50	8.17	8.17	0.26	0.3000
177	30.60	9.09	9.09	0.29	0.3000
178	30.70	7.29	7.29	0.23	0.3000
179	30.80	5.45	5.45	0.17	0.3000
180	30.90	5.43	5.43	0.17	0.3000
181	31.00	6.18	6.18	0.20	0.3000
182	31.10	4.76	4.76	0.15	0.3000
183	31.20	3.30	3.30	0.10	0.3000
184	31.30	2.70	2.70	0.09	0.3000
185	31.40	3.01	3.01	0.10	0.3000
186	31.50	3.18	3.18	0.10	0.3000
187	31.60	3.57	3.57	0.11	0.3000
188	31.70	2.74	2.74	0.09	0.3000
189	31.80	1.89	1.89	0.06	0.3000
190	31.90	1.89	1.89	0.06	0.3000
191	32.00	1.89	1.89	0.06	0.3000
192	32.10	1.32	1.32	0.04	0.3000
193	32.20	1.32	1.32	0.04	0.3000
194	32.30	1.80	1.80	0.06	0.3000
195	32.40	0.90	0.90	0.03	0.3000
196	32.50	1.40	1.40	0.04	0.3000
197	32.60	1.74	1.74	0.06	0.3000
198	32.70	1.01	1.01	0.03	0.3000
199	32.80	1.09	1.09	0.03	0.3000
200	32.90	1.29	1.29	0.04	0.3000
201	33.00	1.29	1.29	0.04	0.3000
202	33.10	1.29	1.29	0.04	0.3000
203	33.20	1.29	1.29	0.04	0.3000
204	33.30	0.48	0.48	0.02	0.3000
205	33.40	0.48	0.48	0.02	0.3000
206	33.50	0.48	0.48	0.02	0.3000
207	33.60	0.98	0.98	0.03	0.3000
208	33.70	0.15	0.15	0.00	0.3000
209	33.80	0.28	0.28	0.01	0.3000
210	33.90	0.03	0.03	0.00	0.3000
211	34.00	0.18	0.18	0.01	0.3000
212	34.10	0.18	0.18	0.01	0.3000
213	34.20	0.18	0.18	0.01	0.3000
214	34.30	−0.80	0.80	0.03	0.3000
215	34.40	−0.80	0.80	0.03	0.3000
216	34.50	−0.80	0.80	0.03	0.3000

217	34.60	−0.80	0.80	0.03	0.3000
218	34.70	−0.80	0.80	0.03	0.3000
219	34.80	−0.72	0.72	0.02	0.3000
220	34.90	−0.32	0.32	0.01	0.3000
221	35.00	0.07	0.07	0.00	0.3000
222	35.10	0.34	0.34	0.01	0.3000
223	35.20	0.34	0.34	0.01	0.3000
224	35.30	0.34	0.34	0.01	0.3000
225	35.40	0.60	0.60	0.02	0.3000
226	35.50	0.60	0.60	0.02	0.3000
227	35.60	0.60	0.60	0.02	0.3000
228	35.70	0.60	0.60	0.02	0.3000
229	35.80	0.60	0.60	0.02	0.3000
230	35.90	0.60	0.60	0.02	0.3000
231	36.00	0.76	0.76	0.02	0.3000
232	36.10	0.89	0.89	0.03	0.3000
233	36.20	0.89	0.89	0.03	0.3000
234	36.30	0.89	0.89	0.03	0.3000
235	36.40	0.89	0.89	0.03	0.3000
236	36.50	0.89	0.89	0.03	0.3000
237	36.60	0.89	0.89	0.03	0.3000
238	36.70	0.89	0.89	0.03	0.3000
239	36.80	0.89	0.89	0.03	0.3000
240	36.90	0.89	0.89	0.03	0.3000
241	37.00	1.03	1.03	0.03	0.3000
242	37.10	1.07	1.07	0.03	0.3000
243	37.20	1.33	1.33	0.04	0.3000
244	37.30	1.33	1.33	0.04	0.3000
245	37.40	0.53	0.53	0.02	0.3000
246	37.50	0.53	0.53	0.02	0.3000
247	37.60	0.53	0.53	0.02	0.3000
248	37.70	0.53	0.53	0.02	0.3000
249	37.80	0.53	0.53	0.02	0.3000
250	37.90	0.53	0.53	0.02	0.3000
251	38.00	0.53	0.53	0.02	0.3000
252	38.10	0.53	0.53	0.02	0.3000
253	38.20	0.53	0.53	0.02	0.3000
254	38.30	0.53	0.53	0.02	0.3000
255	38.40	0.53	0.53	0.02	0.3000
256	38.50	0.53	0.53	0.02	0.3000
257	38.60	0.53	0.53	0.02	0.3000
258	38.70	0.53	0.53	0.02	0.3000
259	38.80	0.53	0.53	0.02	0.3000
260	38.90	0.53	0.53	0.02	0.3000
261	39.00	0.53	0.53	0.02	0.3000
262	39.10	0.53	0.53	0.02	0.3000
263	39.20	0.53	0.53	0.02	0.3000
264	39.30	0.53	0.53	0.02	0.3000
265	39.40	0.53	0.53	0.02	0.3000
266	39.50	0.53	0.53	0.02	0.3000
267	39.60	0.53	0.53	0.02	0.3000
268	39.70	−0.41	0.41	0.01	0.3000
269	39.80	−0.23	0.23	0.01	0.3000
270	39.90	−0.23	0.23	0.01	0.3000
271	40.00	−0.23	0.23	0.01	0.3000
272	40.10	−0.23	0.23	0.01	0.3000
273	40.20	−0.23	0.23	0.01	0.3000
274	40.30	−0.23	0.23	0.01	0.3000

275	40.40	−0.09	0.09	0.00	0.3000
276	40.50	−0.09	0.09	0.00	0.3000
277	40.60	−0.09	0.09	0.00	0.3000
278	40.70	−0.09	0.09	0.00	0.3000
279	40.80	−0.09	0.09	0.00	0.3000
280	40.90	−0.09	0.09	0.00	0.3000
281	41.00	−0.09	0.09	0.00	0.3000
282	41.10	−0.09	0.09	0.00	0.3000
283	41.20	−0.09	0.09	0.00	0.3000
284	41.30	−0.09	0.09	0.00	0.3000
285	41.40	−0.09	0.09	0.00	0.3000
286	41.50	−0.09	0.09	0.00	0.3000
287	41.60	−0.09	0.09	0.00	0.3000
288	41.70	−0.09	0.09	0.00	0.3000
289	41.80	−0.09	0.09	0.00	0.3000
290	41.90	−0.09	0.09	0.00	0.3000
291	42.00	−0.09	0.09	0.00	0.3000
292	42.10	−0.09	0.09	0.00	0.3000
293	42.20	−0.09	0.09	0.00	0.3000
294	42.30	−0.09	0.09	0.00	0.3000
295	42.40	−0.09	0.09	0.00	0.3000
296	42.50	−0.09	0.09	0.00	0.3000
297	42.60	−0.09	0.09	0.00	0.3000
298	42.70	−0.09	0.09	0.00	0.3000
299	42.80	−0.09	0.09	0.00	0.3000
300	42.90	−0.09	0.09	0.00	0.3000
301	43.00	−0.09	0.09	0.00	0.3000
302	43.10	−0.09	0.09	0.00	0.3000
303	43.20	−0.09	0.09	0.00	0.3000
304	43.30	−0.09	0.09	0.00	0.3000
305	43.40	−0.09	0.09	0.00	0.3000
306	43.50	−0.09	0.09	0.00	0.3000
307	43.60	−0.09	0.09	0.00	0.3000
308	43.70	−0.09	0.09	0.00	0.3000
309	43.80	−0.09	0.09	0.00	0.3000
310	43.90	−0.09	0.09	0.00	0.3000
311	44.00	−0.09	0.09	0.00	0.3000
312	44.10	−0.09	0.09	0.00	0.3000
313	44.20	−0.09	0.09	0.00	0.3000
314	44.30	−0.09	0.09	0.00	0.3000
315	44.40	−0.09	0.09	0.00	0.3000
316	44.50	−0.09	0.09	0.00	0.3000
317	44.60	−0.09	0.09	0.00	0.3000
318	44.70	−0.09	0.09	0.00	0.3000
319	44.80	−0.09	0.09	0.00	0.3000
320	44.90	−0.09	0.09	0.00	0.3000
321	45.00	−0.09	0.09	0.00	0.3000
322	45.10	−0.09	0.09	0.00	0.3000
323	45.20	−0.09	0.09	0.00	0.3000
324	45.30	−0.09	0.09	0.00	0.3000
325	45.40	−0.09	0.09	0.00	0.3000
326	45.50	−0.09	0.09	0.00	0.3000
327	45.60	−0.09	0.09	0.00	0.3000
328	45.70	−0.09	0.09	0.00	0.3000
329	45.80	−0.09	0.09	0.00	0.3000
330	45.90	−0.09	0.09	0.00	0.3000
331	46.00	−0.09	0.09	0.00	0.3000
332	46.10	−0.09	0.09	0.00	0.3000

333	46.20	−0.09	0.09	0.00	0.3000
334	46.30	−0.09	0.09	0.00	0.3000
335	46.40	−0.09	0.09	0.00	0.3000
336	46.50	−0.09	0.09	0.00	0.3000
337	46.60	−0.09	0.09	0.00	0.3000
338	46.70	−0.09	0.09	0.00	0.3000
339	46.80	−0.09	0.09	0.00	0.3000
340	46.90	−0.09	0.09	0.00	0.3000
341	47.00	−0.09	0.09	0.00	0.3000
342	47.10	−0.09	0.09	0.00	0.3000
343	47.20	−0.09	0.09	0.00	0.3000
344	47.30	−0.09	0.09	0.00	0.3000
345	47.40	−0.09	0.09	0.00	0.3000
346	47.50	0.00	0.00	0.00	0.3000

Abbreviations: BMI (body mass index) and Lk (log-rank statistic).

Table S2. Characteristics of colorectal cancer patients according to cut-off point of BMI at diagnosis for overall survival.

Characteristics	BMI (kg/m²), SNUH				P^a	BMI (kg/m²), NHID				p^a
	<20.2 (*N* = 913)		≥20.2 (*N* = 4,902)			<20.2 (*N* = 5,462)		≥20.2 (*N* = 48,581)		
	N	(%)	N	(%)		N	(%)	N	(%)	
Age at diagnosis (years), mean (SD)	62.8	(13.2)	62.1	(10.8)	0.10	63.8	(11.9)	62.6	(10.1)	<0.01
Sex					0.36					<0.01
Men	549	(60.1)	3,026	(61.7)		3,331	(61.0)	30,778	(63.4)	
Women	364	(39.9)	1,876	(38.3)		2,131	(39.0)	17,803	(36.7)	
Alcohol drinking status					<0.01					<0.01
Never	670	(73.4)	3,272	(66.8)		2,225	(40.7)	19,889	(49.9)	
Ever	232	(25.4)	1,548	(31.6)		1,806	(33.1)	18,578	(38.2)	
Smoking status					0.07					<0.01
Never	703	(77.0)	3,886	(79.3)		3,019	(55.3)	28,783	(59.3)	
Ever	199	(21.8)	973	(19.1)		2,276	(41.7)	18,400	(37.9)	
Diabetes mellitus					<0.01					<0.01
No	820	(89.8)	4,159	(84.8)		4,561	(83.5)	39,475	(81.3)	
Yes	92	(10.1)	742	(15.1)		901	(16.5)	9,106	(18.7)	
Hypertension					<0.01					<0.01
No	694	(76.0)	3,044	(62.1)		4,058	(74.3)	30,918	(63.6)	
Yes	218	(23.9)	1,857	(37.9)		1,404	(25.7)	17,663	(36.4)	
Tumor site					0.05					0.01
Colon	564	(61.8)	3,196	(65.2)		3,764	(68.9)	34,921	(71.9)	
Rectum	349	(38.2)	1,706	(34.8)		1,496	(27.4)	12,275	(25.3)	
TNM stage					<0.01					
I	173	(18.9)	1,164	(23.8)		-		-		
II	384	(42.1)	1,727	(35.2)		-		-		
III	356	(39.0)	2,011	(41.0)		-		-		
Perioperative chemotherapy					<0.01					0.33
No	309	(33.8)	1,386	(28.3)		4,190	(76.7)	37,552	(77.3)	
Yes	505	(55.3)	2,974	(60.7)		1,272	(23.3)	11,029	(22.7)	
Perioperative radiotherapy					0.65					0.46
No	631	(69.1)	3,365	(68.7)		4,869	(89.1)	43,465	(89.5)	
Yes	185	(20.3)	946	(19.3)		593	(10.9)	5,116	(10.5)	

Abbreviations: BMI (body mass index), SNUH (Seoul National University Hospital), NHID (National Health Insurance Database), SD (standard deviation), TNM (tumor-node-metastasis). [a] Chi-square test for categorical variables and T-test for continuous variables.

Table S3. Association between BMI cut-off point and overall survival in SNUH colorectal cancer patients according to the different multivariable models.

**Characteristics**	**Total**		**Event**		**Age and sex-adjusted model** [a]			**Mutivariable-adjusted model 1**[b]			**Mutivariable-adjusted model 2**[c]		
	N	**(%)**	***N***	**(%)**	**HR**	**(95% CI)**	***p***	**HR**	**(95% CI)**	***p***	**HR**	**(95% CI)**	***p***
BMI at diagnosis (kg/m^2), SNUH													
<20.2	913	(15.7)	265	(22.5)	1.00	(ref.)		1.00	(ref.)		1.00	(ref.)	
≥20.2	4,902	(84.3)	915	(77.5)	0.61	(0.53–0.70)	1.6×10^{-12}	0.62	(0.54–0.72)	1.1×10^{-10}	0.66	(0.57–0.76)	1.8×10^{-8}

Abbreviation: BMI (body mass index), HR (hazard ratio), CI (confidence interval), and SNUH (Seoul National University Hospital); [a] Cox proportional hazard model adjusted for age and sex.; [b] Cox proportional hazard model adjusted for age, sex, hypertension, tumor site, and TNM stage; [c] Cox proportional hazard model adjusted for age, sex, hypertension, tumor site, TNM stage, tumor grade, bowel obstruction, bowel perforation, and ASA grade.

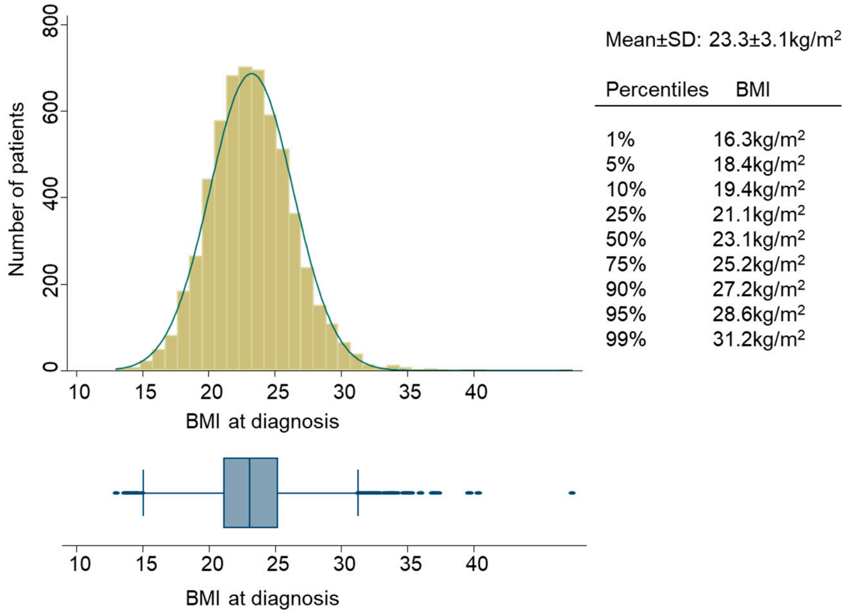

Figure S1. A histogram and a box-plot for BMI at diagnosis in colorectal cancer patients, SNUH. Abbreviations: BMI (body mass index), SNUH (Seoul National University Hospital), SD (standard deviation).

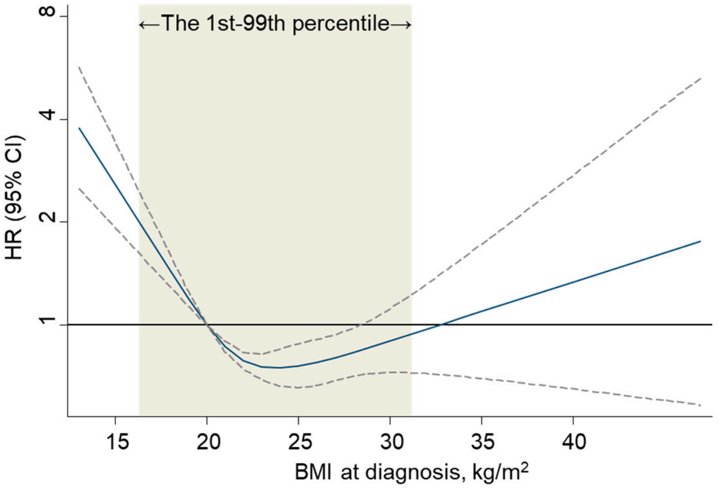

Figure S2. A restricted cubic spline plot of association between BMI at diagnosis and mortality in colorectal cancer patients, SNUH. The number of knots were set at 4. The BMI of 20kg/m^2 was used as reference value in a Cox proportional hazard model adjusted age, sex, history of hypertension, tumor site, and TNM stage. The solid line indicates hazard ratio and the dashed line indicates 95% CI. The range from the 1st percentile (16.3kg/m^2) to 99th percentile (31.2kg/m^2) of the BMI was colored in gray. Abbreviations: BMI (body mass index) and SNUH (Seoul National University Hospital), HR (hazard ratio), and CI (confidence interval).

© 2020 by the authors. Submitted for possible open access publication under the terms and conditions of the Creative Commons Attribution (CC BY) license (http://creativecommons.org/licenses/by/4.0/).
